# MEVA - An Interactive Visualization Application for Validation of Multifaceted Meteorological Data with Multiple 3D Devices

**DOI:** 10.1371/journal.pone.0123811

**Published:** 2015-04-27

**Authors:** Carolin Helbig, Lars Bilke, Hans-Stefan Bauer, Michael Böttinger, Olaf Kolditz

**Affiliations:** 1 Department of Environmental Informatics, Helmholtz Centre for Environmental Research (UFZ), Leipzig, Germany; 2 Faculty of Environmental Sciences, Technical University Dresden, Dresden, Germany; 3 Institute of Physics and Meteorology, University of Hohenheim, Stuttgart, Germany; 4 German Climate Computing Center (DKRZ), Hamburg, Germany; The Ohio State University, UNITED STATES

## Abstract

**Background:**

To achieve more realistic simulations, meteorologists develop and use models with increasing spatial and temporal resolution. The analyzing, comparing, and visualizing of resulting simulations becomes more and more challenging due to the growing amounts and multifaceted character of the data. Various data sources, numerous variables and multiple simulations lead to a complex database. Although a variety of software exists suited for the visualization of meteorological data, none of them fulfills all of the typical domain-specific requirements: support for quasi-standard data formats and different grid types, standard visualization techniques for scalar and vector data, visualization of the context (e.g., topography) and other static data, support for multiple presentation devices used in modern sciences (e.g., virtual reality), a user-friendly interface, and suitability for cooperative work.

**Methods and Results:**

Instead of attempting to develop yet another new visualization system to fulfill all possible needs in this application domain, our approach is to provide a flexible workflow that combines different existing state-of-the-art visualization software components in order to hide the complexity of 3D data visualization tools from the end user. To complete the workflow and to enable the domain scientists to interactively visualize their data without advanced skills in 3D visualization systems, we developed a lightweight custom visualization application (MEVA - multifaceted environmental data visualization application) that supports the most relevant visualization and interaction techniques and can be easily deployed. Specifically, our workflow combines a variety of different data abstraction methods provided by a state-of-the-art 3D visualization application with the interaction and presentation features of a computer-games engine. Our customized application includes solutions for the analysis of multirun data, specifically with respect to data uncertainty and differences between simulation runs. In an iterative development process, our easy-to-use application was developed in close cooperation with meteorologists and visualization experts. The usability of the application has been validated with user tests. We report on how this application supports the users to prove and disprove existing hypotheses and discover new insights. In addition, the application has been used at public events to communicate research results.

## Introduction

For many decades researchers have been working on the development of numerical weather and climate models with the aim to predict future developments. Climate modeling aims to simulate mean values and statistical properties of the climate system for a long time span. In contrast, weather modeling aims to predict the state of the atmosphere for a short time span [1]. Regional weather forecast simulations are based on the latest observations such as station, radar, and satellite data as well as on interpolated results of global models and older model predictions that serve to provide as accurate as possible initial and boundary conditions for the simulation [2]. To perform these predictions, modelers define the atmosphere as a fluid and simulate its state [1].

Meteorologists proceed in developing weather and climate models with ever increasing resolution and complexity. This results in the generation of larger and larger data sets. In order to reduce uncertainty in the predictions, it is common today to perform ensembles of simulations: multiple model runs are done with slightly varying initial conditions. With a statistical analysis of the resulting ensemble data, the probability of specific model results can be determined. The ensemble data is another source of the strong data growth in this discipline.

For the visual analysis of weather simulations, it is mandatory to display the data together with its geographical context (e.g., time-independent data such as topography, cities, and water bodies), since some meteorological phenomena can be correlated with these data. Furthermore, the integration of relevant observational data is necessary to visually compare this information with the simulation results and to identify relationships and inconsistencies. Consequently, meteorologists are faced with exploring and analyzing data sets from different sources *(multimodal)* that are very complex. Those data sets consist of *spatiotemporal* data and include numerous variables *(multivariate)*. In addition, data sets with multiple model runs *(multirun)* have to be analyzed [3]. To explore, analyze and present such data, scientific visualization methods for multifaceted data have to be developed [4–6].

Together with experts in meteorology and visualization, we develop a workflow and an application that can cooperatively be used to verify, falsify and generate hypotheses, study phenomena and their evolution, discover inconsistencies and unexpected features in the evolution and representation of processes in the atmosphere. We combine standard 3D visualization methods for time-dependent meteorological data and implement a visualization application with a graphical user interface (GUI) that supports users in analyzing these data sets. To tackle this challenge, we pursue an approach that combines the power and variety of data abstraction methods of a visualization tool (ParaView [7]) with the interaction and presentation capabilities offered by computer-games engines (Unity [8]).

To import the data sets into the visualization software, the data has to be converted (e.g., WRF netCDF data has to be converted to fit the netCDF conventions supported by ParaView) so that it can be incorporated by the visualization software. In addition, inconsistencies (e.g., differences in scale, resolution, coordinate system) between the data sets have to be identified and removed with the help of the visualization software or by prior processing steps [9]. To enable the user to analyze the data by using state-of-the-art visualization techniques (e.g., isosurfaces, streamlines with custom seeding, path lines, individual glyphs or calculation of variables before adding a visualization filter) and to be able to cooperatively analyze the data, the application needs to support multiple presentation devices that are used in modern sciences (e.g., virtual reality environments, head-mounted displays). Usually meteorologists are not using complex visualization methods in their daily work. Hence, the application should have an easy-to-use interface based on the needs of the user to enable him to quickly orient himself.

There are many visualization tools specifically developed for spatiotemporal, multivariate weather and climate data [10], but they usually offer only very limited support for multirun and multimodal data [3, 11]. Furthermore, most tools support just basic visualization methods (e.g., simple streamlines, isolines). To analyze phenomena like turbulence in high resolution simulations, it is necessary to provide a variety of more complex 3D visualization methods. Therefore representations such as 3D streamlines with high density, trajectories or path lines can be used.

In our case study, the input data sets are quite large (e.g., one time step with a size of 358 MB) which can cause problems with the performance in the visualization application. When applying visualization methods (e.g., isosurfaces) the original data is filtered. Usually, the data size of the resulting geometries is very small in comparison to the input data (e.g., geometries of one time step with eight variables has a size of 1.3 MB). Based on the visualization methods and parameters selected, 3D objects are generated.

Existing visualization applications include very sophisticated methods and parameters. For Example, the calculation of wind fields requires the application of a streamline algorithm including the integration of appropriate seed points. These methods are cumbersome for people who do not use visualization software in their daily work. To enhance the usability for end users, it is necessary to provide a simplified user interface that is well adapted to their needs and skills.

The weather simulation results consist of 3D data including 3D scalar and 3D flow data. Therefore, it is necessary to support advanced 3D visualization devices in order to deal with the high dimensionality of multifaceted data. As an example, virtual reality environments are a promising tool to visualize such complex data sets [12, 13]. A visualization application should support cooperative work because nowadays it is common for scientists to work closely together on the same project while they are spatially separated from each other.

We present an application, which is developed in close cooperation with meteorologists and visualization experts, that fulfills the described needs. Therefore, we developed a workflow based on the visualization software ParaView and the computer-games engine Unity to deliver a portable and easy-to-use visualization application together with the pre-computed geometries for the case study. We offer methods for displaying and comparing different simulation runs side-by-side or with outlines (marking the borders of geometries) and change settings of 3D objects (e.g., opacity). The application is portable to different platforms; hence, it can easily be used in discussions, presentations, and even for educational purposes. The use of the application is not restricted to weather data. It is usable for all kind of environmental data such as groundwater simulation results and geological models.

Finally, the usability and benefits of the application are evaluated with user tests.

## Related work

The visualization of research results has a growing importance in nearly all scientific domains (e.g., environmental research [9, 14, 15], genetics [16, 17], biomedicine [18], and animal movement [19, 20]). We provide an overview of related work with a focus on visualization applications for earth system sciences, visualization applications in general, using movie applications for presenting, and approaches for using computer-games engines for scientific visualization.

### Domain specific visualization applications for earth system sciences

Since data from weather and climate simulations are quite large and complex, many visualization applications have been developed especially for this application domain. An overview is given by Nocke et al. [10]. One of the first applications specifically developed for meteorological model data is Vis5D [21] in 1988. Unfortunately, the development ended in 2002, but Vis5D is still used by many meteorologists. A more recent example is VAPOR [22], a visualization application developed especially for *Weather Research and Forecast model* (WRF) simulation data, which is the model used in our case study (see Meteorological background). Another approach is to integrate climate and weather data into web-based applications like Google Maps and Google Earth [23, 24].

All these applications allow for the visualization of weather simulations based on single model runs, and are mainly restricted to only a few data types common in weather and climate research (as NetCDF, HDF or GRIB). But, because of its huge relevance in climate and weather research, the visualization of multirun and scenario-based simulations is also an important challenge. Domain specific visualization applications such as Noodles [25] support multirun data, but unfortunately are restricted to 2D data and just take one or two different variables into account [26]. In general, the visualization methods used in these applications are very basic. The data types that could be imported are restricted to those common in weather and climate simulation research (mostly netCDF).

### General purpose visualization applications

Scientific visualization is applied in a broad range of application fields, ranging from molecular to medical to outer space research. There are some open source (e.g., ParaView [7], VisIt [27], OpenDX [28]) and commercial (e.g., Avizo [29]) general purpose 3D visualization systems available that meet domain specific requirements and provide some of the newest visualization methods and algorithms. However, due to the variety of operations and parameters offered by the systems, their user interfaces are quite complex and it is quite challenging and time-consuming for non-visualization experts to familiarize themselves with the different systems and produce meaningful visualizations of their data.

### Movie applications

Video technology is used to overcome lacking interactivity in the visualization process in particular for large, time-dependent data. Using movies to publish visualizations especially designed with respect to a broader public is another approach which we also used in our previous work [30]. The production of complex videos can be very time-consuming because of the large development effort [31]. A screenplay has to be developed, camera paths have to be generated in synchronization with the time animation of the data, and the resulting sequences need to be captured and finally edited. The visualization developer has to identify the most effective camera positions and settings, the most appropriate parameters for the visualization and for how long a specific view should be available. This is necessary because besides the player controls, no interaction methods can be provided to the user. An advantage of this approach is the platform independence: movies can be played back on various different devices and over the Internet. Depending on the rendering, it is also possible to export stereoscopic videos meeting the requirements of 3D players.

### Game applications

The reasons for using a computer-games engine for scientific visualization tasks are manifold. For example, they allow for rapid prototyping: the desired application functionality can be easily integrated with the help of visual editing and scripting, and a strong background in software engineering is not necessarily required. Their support of a variety of presentation devices such as PCs, game consoles and mobile devices [32] is advantageous. Furthermore, they enable access to one of the most sophisticated rendering pipelines that can be found [33]. But up to now only a few examples demonstrate the use of computer-games engines for scientific visualization (e.g. molecular structures using the Unity engine [31], cave reconstructions with the Quake3 engine, terrain shaping and vegetation placement with the Far engine [34] and prediction of protein structures with Foldit [35]).

## Workflow and application

**Fig 1:**
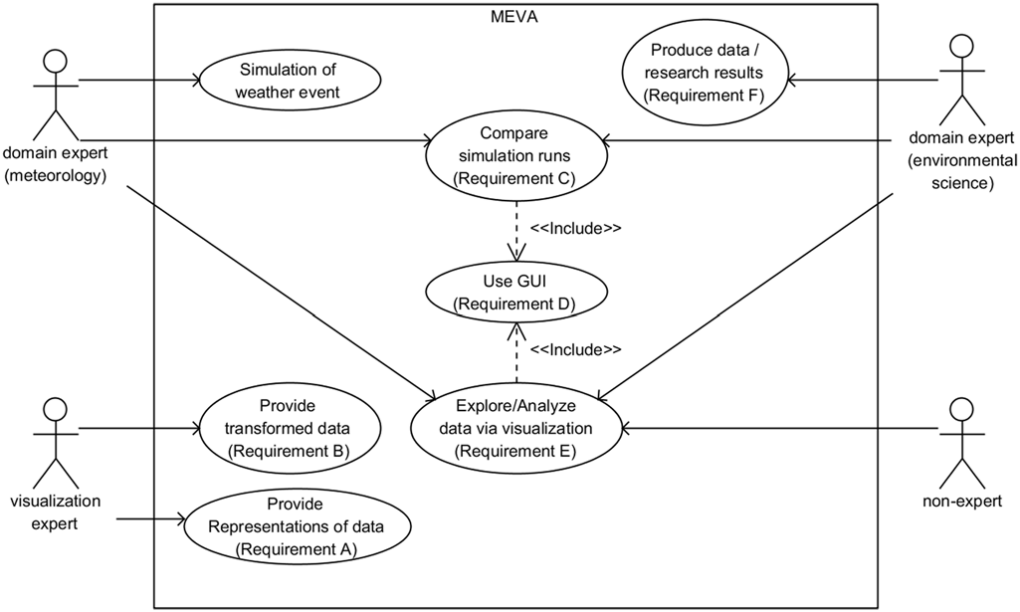
Use case diagram. Together with domain experts, we specified a set of requirements that are the basis for the development of the workflow and the application.

At the beginning of our work, we specified a set of requirements together with domain experts. We summarized them in the use case diagram shown in Fig 1. These requirements relate to the gaps we defined in the introduction and will be presented in the following. For the development of our application, we followed an iterative process consisting of designing, testing and prototyping/implementation phases. We defined three workflow steps: (1) data integration, (2) abstraction and representation, and (3) interaction and presentation. As a result, we developed MEVA, an easy-to-use application that provides interaction methods for a large variety of data. We provide MEVA and the scripts used to build our application on our GitHub website [36]. Data of the simulation results and of the generated geometries are available through this repository, too.

### Requirements

#### A: Apply advanced 3D visualization methods

The first requirement we defined is to support most standard 3D visualization methods as well as advanced methods and algorithms such as streamlines or trajectories for vector quantities. The multivariate character of the data requires these methods to find representations for all variables that are intuitive, capable of being differentiated, avoid clutter and occlusion, and are aware of outliers and important patterns [3, 37]. In terms of glyphs, attributes like shape, size, and color can be used to represent different variables and their values. It is also necessary to utilize characteristics of spatiotemporal data like “near things are more related than distant things” [38] and even use space between representations to encode additional information [3]. The fact that the data is not only multivariate, but also multimodal and multirun underscores the importance of using these possibilities. Therefore, it is crucial to place careful consideration into the representation methods of the data, such as side-by-side display.

#### B: Integrate static data, observations and simulation data

The data we take into account is spatiotemporal (x,y,z,t) and consist of the following types:

- High resolution **simulation data** that is the result of different model runs *Sim*_*a*_ (a = 1 … n model runs) with time step size Δ*t*_*a*_ (dependent on the simulation run) and including numerous variables *Var*_*i*_ (i = 1 … n variables, e.g., temperature, wind vector components, mass fraction).
- **Observation data** *Obs* with time step size Δ*t* and including numerous variables *Var*_*j*_ (j = 1 … n variables, e.g., measured surface temperature, amount of precipitation).
- **Static data** *Stat* (time-independent) that includes numerous variables *Var*_*k*_ (k = 1 … n data set consisting of cities, rivers, borders, orography, land use characteristics, etc.).

We aim to combine these data in an effective way.

#### C: Compare experiments (multirun data)

Our objective is to provide visualization and interaction methods to deal with different simulation runs. Therefore, we implement split-screen views and the possibility to distinguish the different runs by marking them with colored outlines. Uncertainty can be displayed by overlaying the runs in one view and manipulating the opacity of each individually. Also, the comparative visualization of data with different temporal and spatial resolution needs to be supported.

#### D: Deploy visualization with easy-to-use GUI

We aim to deploy a graphical user interface (GUI) that is intuitive, self-explanatory and as slim as possible (limited functionality) to avoid overburdening the user. On the other hand, the user needs to be able to interact with the visualization: to move the camera, to select subsets of variables for display, to change the opacity of their visual representations, to control the time animation, or to additionally blend in a 2D overview map. To get an impression of values changing over time, we need to generate animations at constant frame rates.

#### E: Support for 3D presentation devices

In order to reduce the technical restrictions for using the visualization application to a minimum, it should work on a variety of different platforms, including different 3D presentation devices such as virtual reality environments, head-mounted displays, 3D projectors, and desktop PCs. The scientists can use it for analysis of their results in order to gain more insight and even arrange for interactive collaborative walkthroughs with colleagues and project partners [31]. In addition, one aim addressed by this requirement is to inform the public by providing an application for edutainment [34, 39].

#### F: Support for various environmental data

The application should also be usable with other environmental data, different spatial and temporal resolutions or coordinate reference systems. By using modular code and providing scripts for displaying variables, time controls, camera controls and an overview map, a basis application for visualization projects in environmental sciences can be provided.

### Development and implementation of the visualization workflow

#### Data integration

**Fig 2:**
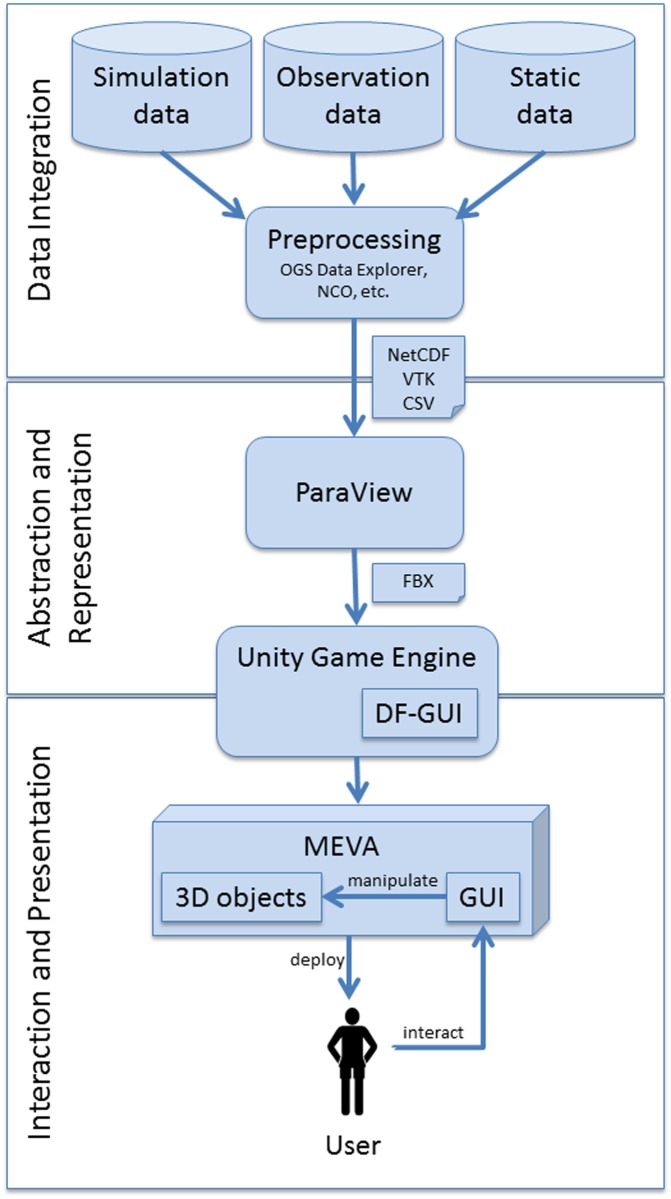
Workflow leading from input data to visualization. The data integration includes preprocessing methods and is followed by the abstraction and representation step that prepares the data for the game engine Unity. In the final interaction and presentation step, the application is deployed to the user and he can interact with the visualization using the GUI and manipulate the 3D objects.

According to requirement B, it is necessary to provide data integration methods to transform input data into a format readable by the visualization application. We used open source tools like NCO and the OpenGeoSys (OGS) Data Explorer [9, 40] (cf. Helbig et al 2014 [30]) for this preprocessing step (see Fig 2).

#### Abstraction and representation

The data sets in environmental research are often very large, especially if 3D simulations are involved. To deal with such large data sets (e.g., with regard to the performance of the visualization), it is obligatory to reduce the data size in a first step (abstraction): e.g. by downsampling (spatially, temporally), selection of subsets, or selection of variables. We worked together with our domain experts to find appropriate abstraction methods.

At first, a minimal subset of the variables required for the analysis is defined. The selection of the subset is done by the domain experts and based on the specific phenomena they want to analyze. It includes variables such as the three wind components, various mass fraction components (e.g., ice, graupel), and temperature. The further abstraction is realized by applying advanced visualization methods (cf. requirement A) to the data sets. The visualized variables for all time steps are then exported in the form of time series of geometries for each variable.

We used the open source visualization software ParaView, which provides a wide range of visualization methods. It also gives us the opportunity to implement our own methods, e.g., using Python scripts. ParaView is well documented and supported by a large, substantial community. In order to create an interface from ParaView to the Unity game engine, we implemented a module for exporting the resulting geometries (e.g., streamlines, isosurfaces) in the widely-used FBX format [8], including meta-data like color scale and range of values [41].

#### Interaction and presentation

In order to fulfill requirements D and E, we decided to use a computer-games engine as a technical basis for the final visualization presentation application. The idea of using a computer-games engine was already discussed by Rhyne in 2000 [42]. It provides several advantages like state-of-the-art graphics, automatic support of major platforms, and low costs (only the developers need to license the software, while the end users can get a free executable to run the application) [31, 33]. We decided to use the computer-games engine Unity because it is freely available and well documented. There are many additional *assets* available that can be integrated into the application. Thus, implemented functionality (e.g., for the GUI, for camera) can be used without investing significant effort into its implementation. In addition, the computer-games engine Unity has a large support community. Besides these advantages, one challenge of using a computer-games engine such as Unity for scientific visualization is the high development effort in the beginning including implementation of basic functions (e.g., for data import).

We implemented an import interface that can handle the metadata using C#. For the presentation of the visualization in a virtual reality environment, it is required to use a GUI that supports multiple displays. The standard GUI included in Unity does not support that. We decided to use the Daikon Forge GUI [43] and implemented elements for objects, time, cameras and overview map. To manage the different data sets, we developed and implemented a hierarchy that can handle multiple data sets that differ in their different temporal resolution and display them in parallel.

### Application interface

We implemented our interface using the computer-games engine Unity and provided the resulting executable to the users. The application shows the current scene and GUI elements, which are collapsible in order to save screen space.

**Fig 3:**
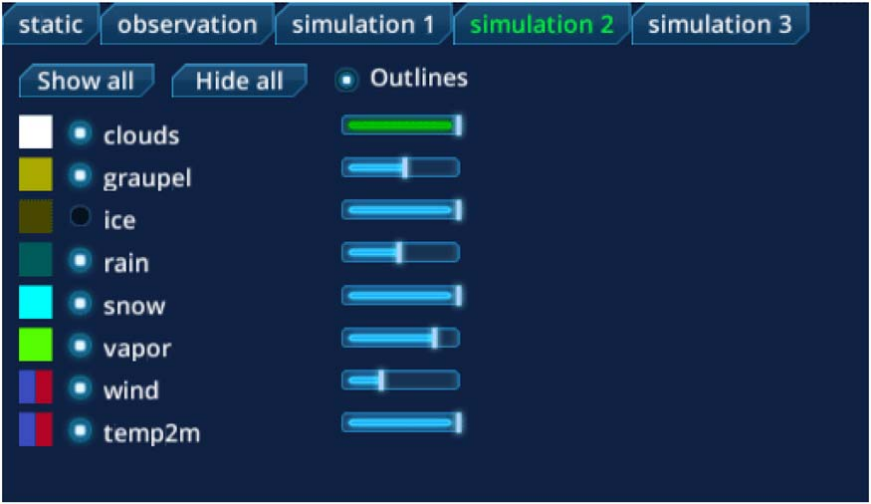
MEVA GUI objects element. With this interface the user can show or hide variables, change their opacity and add outlines to the 3D objects. The variables are grouped according to their type (static data, observation, simulation run a, simulation run b, etc.).

During the development process, we discovered the demand of the user to show and hide single variables and groups of variables. Therefore, we implemented a GUI element (see Fig 3) in which all variables are assigned to a tab according to their type of data (static data, observation data, simulation run a, simulation run b, etc.). Every variable has an assigned radio button to toggle the according 3D objects and a box showing the color code of the 3D objects of the variable. In addition, users have the option to change the opacity of the 3D objects (e.g., reduce the opacity of a cloud to examine the wind circulation inside of it). The combination of variables of various types that are displayed can be defined individually by the user. Furthermore, we also provide the option to add outlines to the 3D objects to distinguish various simulation runs.

We implemented several camera modes that differ in their behavior: orbit (rotating around the center) and split-screen (side-by-side screens for displaying different simulation runs). The camera can be changed using a defined key (spacebar). For the orbit camera, we implemented a script that enables the user to change the position of the camera while rotating it around a predefined point (in our case, the case study area’s center). This functionality is very common in computer games, and there are several functions provided by Unity to implement it (e.g., “RotateAround” function). We decided on this camera type because it allows the user to easily examine the visualization scene. The experiences with other camera types (e.g., first person controller camera that provides ego perspective) showed us that the user runs the risk of getting lost in the scene without the capability to reset to an appropriate perspective. For the split-screen camera we employ two cameras that use the same position but show different layers. A layer in Unity is commonly used to render only a part of a scene. In our case study, each simulation run is assigned to a separate layer.

**Fig 4:**
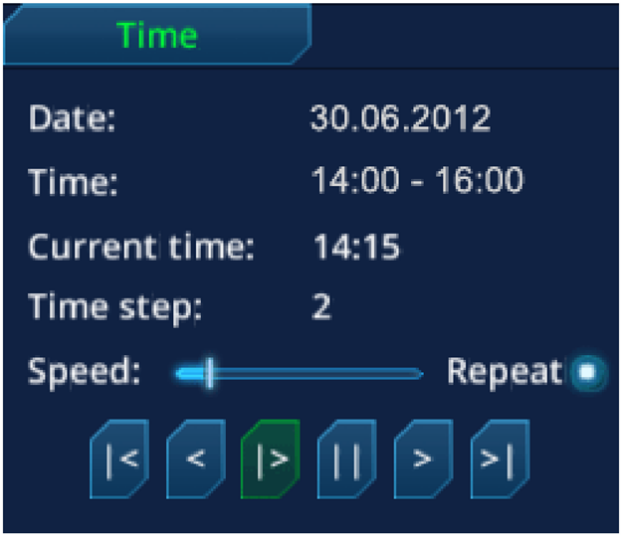
MEVA GUI time element. The interface for the time provides controls such as play, pause, one step forward/back, go to first/last time step. In addition, the user can adjust the speed of the animation.

The user can control the animation of the time steps with the GUI element time (see Fig 4). This component is implemented to handle groups of variables with different time step increments (e.g., simulation 1 with 15 minutes, simulation 2 with 5 minutes). The GUI includes the standard controls (play, pause, one step forward/back, go to first/last time step), and a slider to regulate the speed.

**Fig 5:**
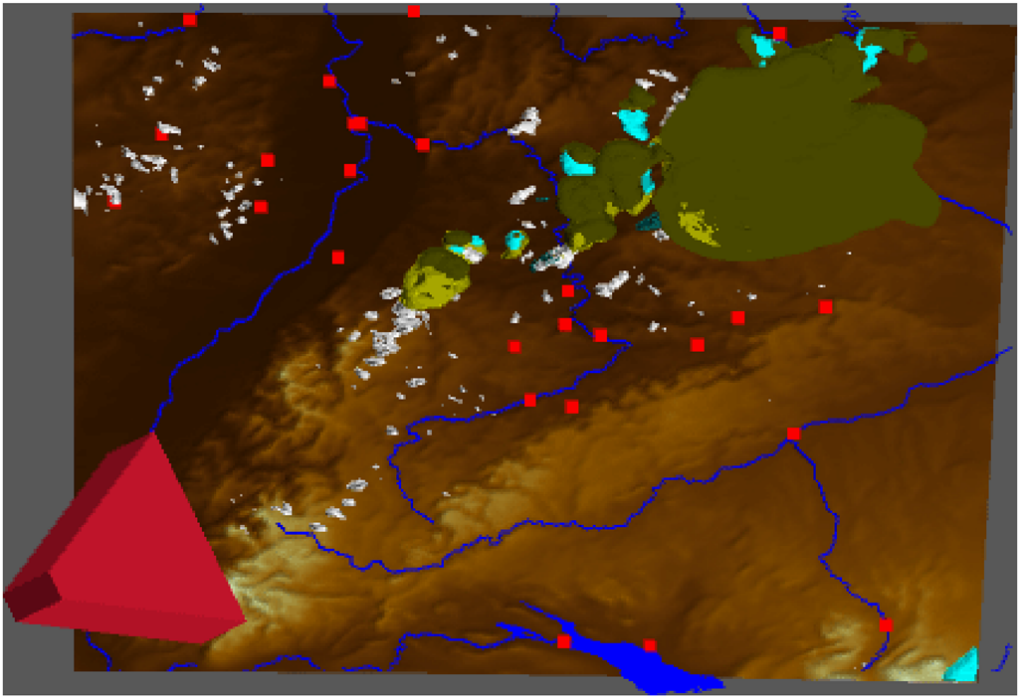
MEVA GUI overview element. For large case study areas, we provide a function for displaying a small overview map in which a polyhedron shows the position and angle of the camera.

In addition, there is a small overview map for orientation that shows the top view of the current scene and includes a polyhedron showing the position and angle of the camera perspective (see Fig 5). This script should be included especially for case studies with large areas where the orientation is challenging.

## Case study

For our case study, we worked in a team of visualization experts and experts in meteorology. The case study area is Baden-Württemberg, a federal state of Germany, where we selected a subset area of about 40 x 35 km. This is the area where the specific meteorological phenomenon, which is described in the next section, appears within the simulation. We took into account a set of simulation data at different horizontal resolutions, observation data and static data, and defined a number of tasks that have to be solved with our approach. Finally, we validated the results together with the experts.

### Meteorological background

In our case study, we focus on simulation data of the limited area model (LAM) *Weather Research and Forecast model* (WRF) [1, 44]. WRF is a state-of-the-art numerical weather prediction model. Supported by a large community, new features and applications are added quickly. The model can be applied for research purposes as well as for operational numerical weather prediction on a wide range of spatial and temporal scales. A large variety of physical parameterizations of different complexity and capabilities to ingest observations by data assimilation are provided by the model. Hence, the model can be applied for numerical weather forecasts, for downscaling of global climate simulations as well as and for detailed studies of small scale meteorological processes, so-called large-eddy-simulations (LES) with grid increments on the order of 100 m.

MEVA is tested with the data from an interesting high-impact weather situation. In the area of interest, the 30th of June, 2012, was among the most significant summertime severe weather days of recent years. To the east of a trough over the British Isles, warm, moist air from the Mediterranean was transported to southern Germany. There had been almost no cloudiness in the morning hours and strong irradiance heated the ground surface to temperatures of 30 degrees C and higher. High temperatures and high moisture destabilized the atmosphere and prepared the environment for the development of severe convection.

Starting from 12 UTC, along a line from the northern Black Forest to the northeast, isolated severe thunderstorms, so-called supercells, developed. A supercell is the most rare of the four types of thunderstorms (single-cell, multi-cell, squall-line and supercell). At the same time, it has the largest potential to produce severe weather ranging from severe precipitation and hail to tornadoes. A deep and constantly rotating updraft, the so called mesocyclone, characterizes supercells. Thereby, a constant provision of energy to the system is guaranteed (with updraft and subsequent condensation). In contrast to single-cell thunderstorms the supercell can be active for hours.

One cell that developed near Pforzheim rapidly increased in strength, reaching radar reflectivity values of up to 60 dBZ within 30 minutes. Near Heilbronn, a cell-splitting—typical for long-lasting rotating thunderstorms—took place. While the left part of the cell died out quickly, the so-called right-mover strengthened and deviated to the right from the main wind direction. Later on, the cell produced hail stones with diameters up to 9 cm near the city of Feuchtwangen in Bavaria.

The situation was selected since the correct representation of especially isolated severe convection is a challenging task in numerical weather prediction. To be able to investigate the capability of the WRF model to represent the development, a downscaling from a global meteorological analysis was set up. The analysis forced the outer WRF domain with 3 km horizontal resolution. Two more domains with 1 km and 333 m horizontal resolution were nested into the outer domain and allow the investigation of the representation of the cell with different horizontal resolutions.

### Tasks

To test and evaluate our application, we defined four tasks at two levels: analysis (task 1 and 2) and presentation (task 3 and 4). In the following, we present them and their results.

#### Task 1: Analyzing the simulated circulation and mass fraction in a supercell with different horizontal resolutions

**Fig 6:**
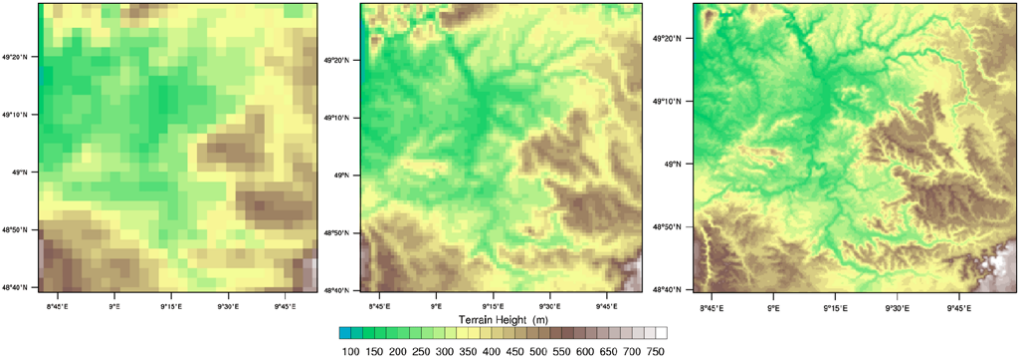
Topography of the subset. The domains have different horizontal resolutions: domain 1 with 3 km, domain 2 with 1 km, and domain 3 with 333 m. Thus, the topography of the region is represented differently in the simulations.

The presentation of multirun data is challenging because of its complex character including space (3D), time, and a set of variables for each simulation run [3] which have to be represented avoiding clutter and occlusion [37]. Different simulation runs are important in the process of developing models and help to identify systematic errors in the model as well as in the parameterization schemes [45]. Therefore, the modelers want to analyze differences between the runs. As described in Meteorological background, we want to analyze the simulated circulation in a supercell as well as the distribution of different hydrometeor mass fractions of three simulations with different spatial and temporal resolutions. Fig 6 shows the topography of the three model runs with the following resolutions:

- Domain 1 with 3 km horizontal resolution and 15 min output interval
- Domain 2 with 1 km horizontal resolution and 5 min output interval
- Domain 3 with 333 m horizontal resolution and 5 min output interval

For our case study, we define subsets of the original data sets in order to focus on the region of the supercell. Here, we visualize the mass fractions by isovolumes and the circulation with streamlines. For the comparison of the multirun data, we provide two approaches: side-by-side and overlay. For the first one, the screen can be split horizontally or vertically to visualize the different runs in separate canvases, but with the same viewing settings. The overlaid comparison uses outlines with a predefined color for every run to distinguish between the different runs. For the streamlines, the color is adapted according to the outline color. The user can adjust the opacity of each variable to create visualizations that suit his needs. At the same time, the overlapping of the isovolumes represents the uncertainty in the variable.

**Fig 7:**
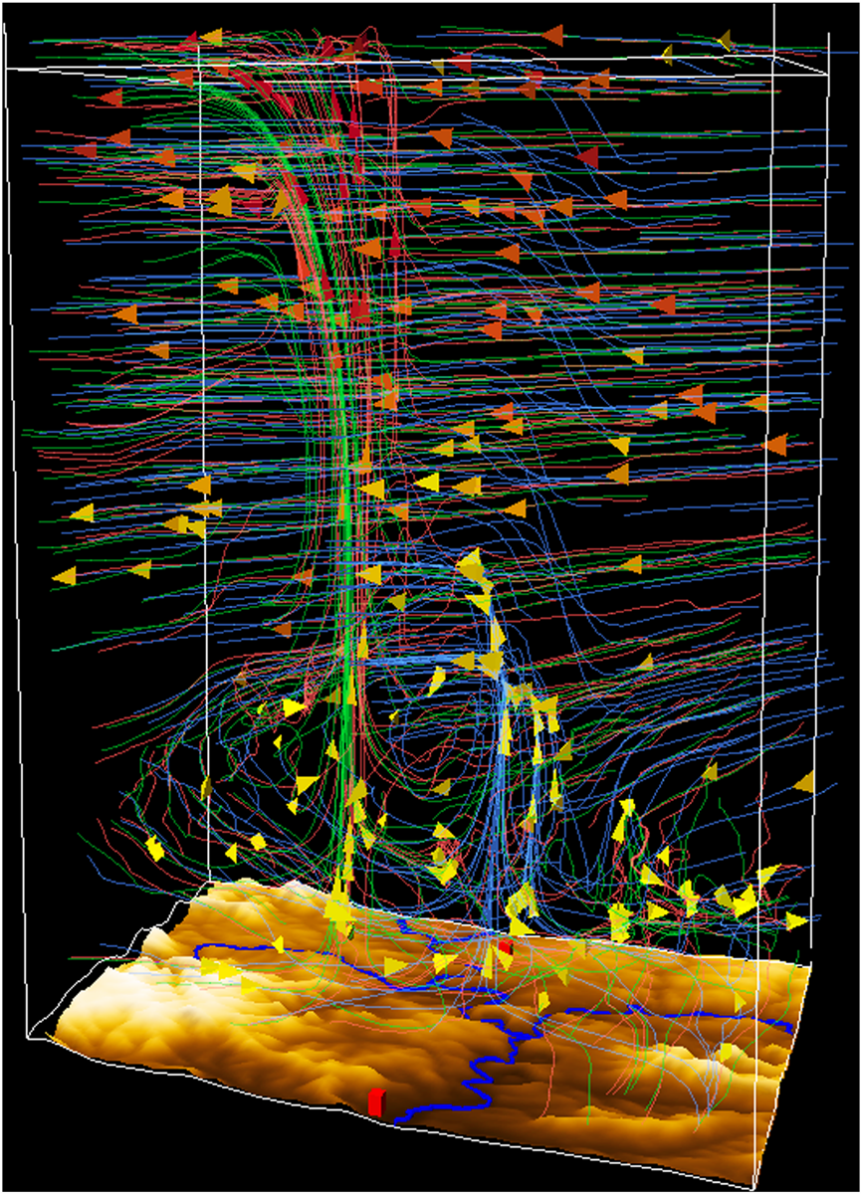
Overlaid view of all domains showing the wind. Domain 1 with 3km resolution (blue), domain 2 with 1km resolution (green), and domain 3 with 333m resolution (red). The state of the streamlines at 2:00 pm is shown using cone glyphs to represent the wind direction. Domain 2 and 3 produced similar wind fields, whereas in domain 1 the upstream is north of the ones in the other domains and not as high as the others. (View towards the south)

Fig 7 shows the overlaid view of the streamlines for all domains: domain 1 (blue), domain 2 (green), and domain 2 (red). The cone glyphs show the wind direction and their colors represent the wind speed. The simulation runs of domain 2 and 3 generated similar wind fields, whereas in domain 1 the updraft is shifted to the north (in the image right). Furthermore, the updrafts in the 1 km and 333 m simulations penetrate higher into the troposphere.

As Fig 7 illustrates, a resolution of 3 km is too coarse to realistically represent the circulation details in the supercell. The broad and more diffusively represented updraft core splits into several branches and does not penetrate through the whole troposphere as expected by conceptual models of developing supercells [46, 47]. This suggests that other internal processes (e.g., stronger lateral inflow into the updraft at higher levels) are acting at that resolution. The representation of the updraft region is much more realistic in the two higher-resolution simulations. In both, the updraft is localized and penetrates to the top of the troposphere. Also, the leeward bending in the upper-tropospheric anvil region of developing thunderstorms is well represented.

**Fig 8:**
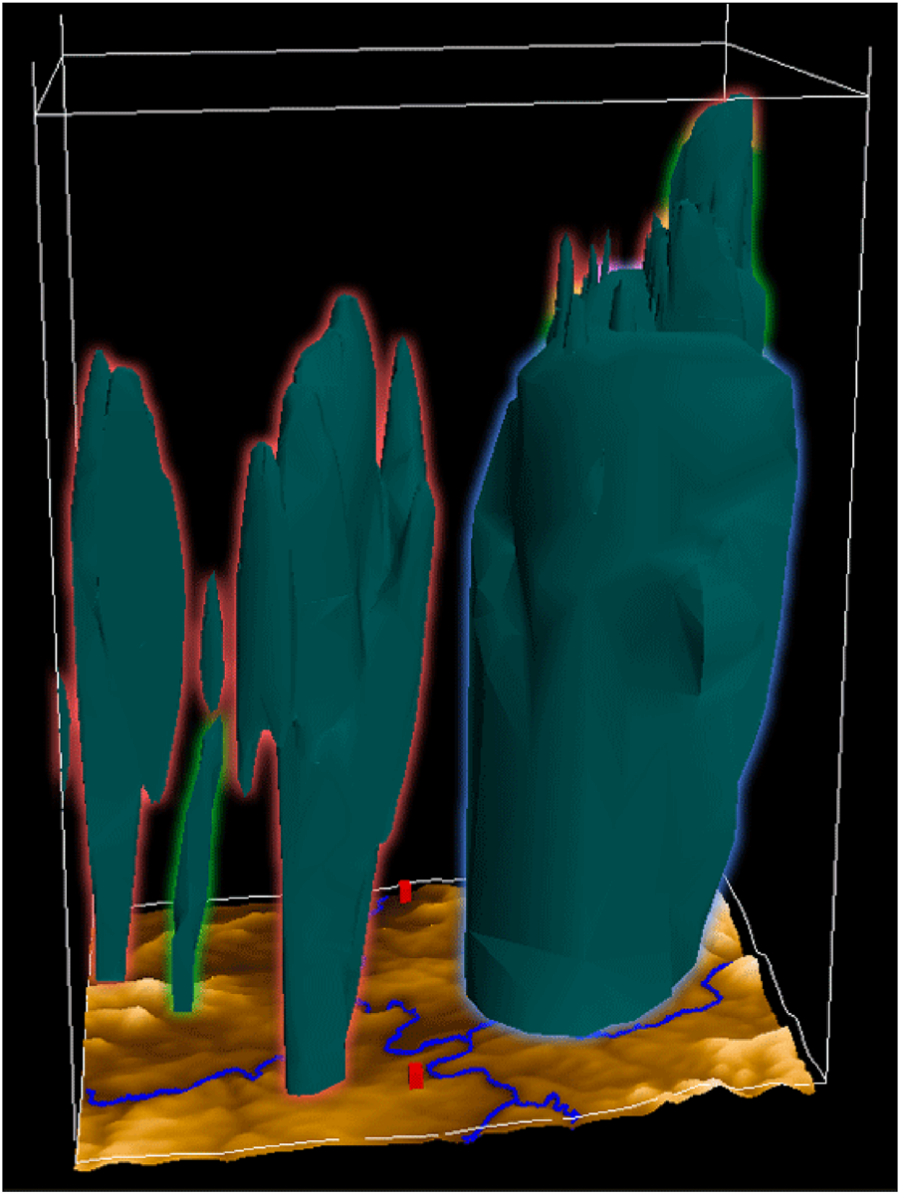
Overlaid view of all domains showing the rain. To distinguish between the different domains, they are marked with outlines: domain 1 with blue, domain 2 with green, and domain 3 with red. It shows the state of the mass fraction of rain at 2:20 pm. The domains with higher resolution produce finer structures in the mass fraction of the rain. (View towards the north)

In Fig 8, the mass fraction of rain is shown in the overlaid view using outlines to distinguish the domains. It can be seen that the domains with higher resolution produce finer structures in the mass fraction of the rain. The rain in domain 2 (green) and domain 3 (red) appears in small cells west of the region (in the image on the left-hand side). In the east, a large ground area is covered by rain in domain 1 (blue). On top of that, rain in domain 2 and 3 are overlapping. The simulation of finer structures with higher resolution is expected to a certain degree. However, it has to be investigated in detail whether the spatial scales of the simulated structures are not underestimated by the higher resolutions. If this is the case, a tuning of the cloud microphysics parameterization might be necessary to lead to an even better simulation.

The large cell is captured by all three simulations. Whereas the 3 km simulation shows a flat top of the precipitation region, the influence of the stronger updraft cores in the finer-scale simulations is illustrated by an overshooting cloud top, reaching higher in the 333 m simulation. This also corresponds to conceptual models of developing thunderstorms.

**Fig 9:**
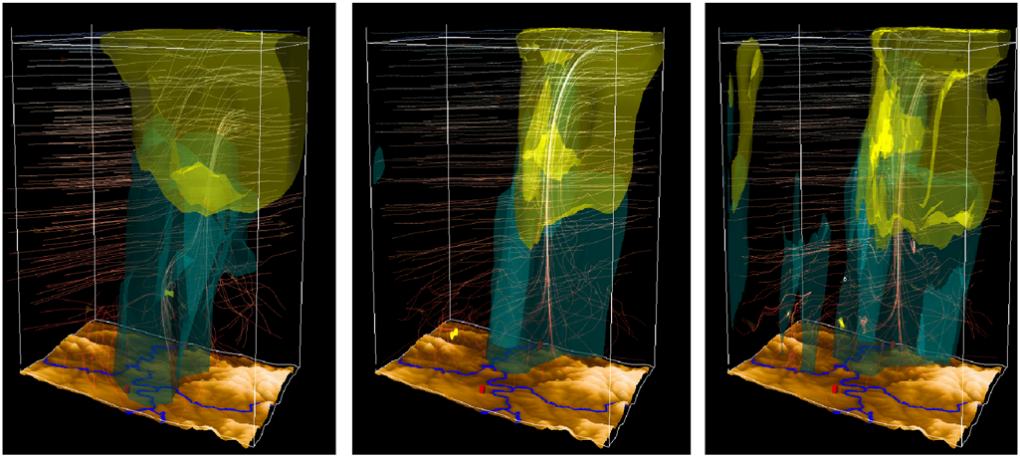
Side-by-side view of all domains. The mass fraction of graupel (light green) and rain (dark turquoise) is shown for all domains (left to right: 3km, 1km, 333m) at 2:00 pm. The finer the resolution of the simulation, the finer the appearing structures in the mass fraction. (View towards the north)

To look at several variables of all domains at the same time, the side-by-side view is used as shown in Fig 9. Here it becomes apparent that the finer the resolution of the simulation, the finer are the appearing structures in the mass fraction, here graupel and rain. The upstream wind field in the western part of the domain is similar in all domains, but differs a little in its location. Especially in the lower troposphere, differences between the resolutions are clearly visible. In contrast to domain 2 and 3, where the updraft is (as expected from conceptual models) one concentrated column in the two higher-resolution simulations, the stream in domain 1 includes a curve. This leads to the hypothesis that different processes are occurring.

An interesting difference between the three visualizations is the separation of the central updraft region and the area of main precipitation. This is an expected feature in a supercell thunderstorm and contributes to its long lifetime by avoiding evaporation and cooling in the central updraft. In the 3 km simulation, the updraft is embedded into the precipitation region. Therefore, evaporation of precipitation might influence the development of the updraft.

The representation improves in the 1 km simulation (domain 2). The central updraft and the region of precipitation are separated. But the resolution is too coarse to simulate internal structure in the precipitation and anvil regions.

In the 333 m simulation (domain 3) more structure is seen: turbulence in the region of the anvil base and in the swirling of the rain area around the precipitation-free updraft region (mesocyclone).

The visualization setup demonstrated its capabilitiy to support the verification of the representation and evolution of atmospheric processes in model simulations. Furthermore, the better view on the relations of different atmospheric variables support the improvement of process understanding, the necessary first step to improve the model physics. The simulations show the importance to operate the model with a fine horizontal resolution for detailed process studies of interesting atmospheric phenomena.

#### Task 2: Comparing observation and simulation data

An important task for the evaluation of model simulations is the comparison with observation data and static data, where users can examine correlations and inconsistencies between them. To manage that, we developed methods for the conversion of the different data sets with their different spatial and temporal resolutions, and implemented export and import interfaces. The integrated data includes observation data from stations of the German Weather Service (DWD) inside the focused region, providing data, e.g., temperature (four stations) and wind measurements (two stations) every hour. In addition, static data like the river network and the location of cities are included. Unfortunately, the observational station data is spatially and temporally very sparse compared to the model data.

We compare the data using visual data fusion: the different data types are overlaid in the visualization. The static data is represented using intuitive representations (e.g., tubes for rivers, boxes for cities) positioned on top of the digital elevation model (DEM). The observation data is represented by glyphs, where the color represents the values. For example, the temperature at a station is represented by the color of a sphere, and the wind is represented by an arrow showing the local wind direction and colorized according to the speed. For quick comparison, the color ranges of the observation data matches the color ranges of the simulation data.

**Fig 10:**
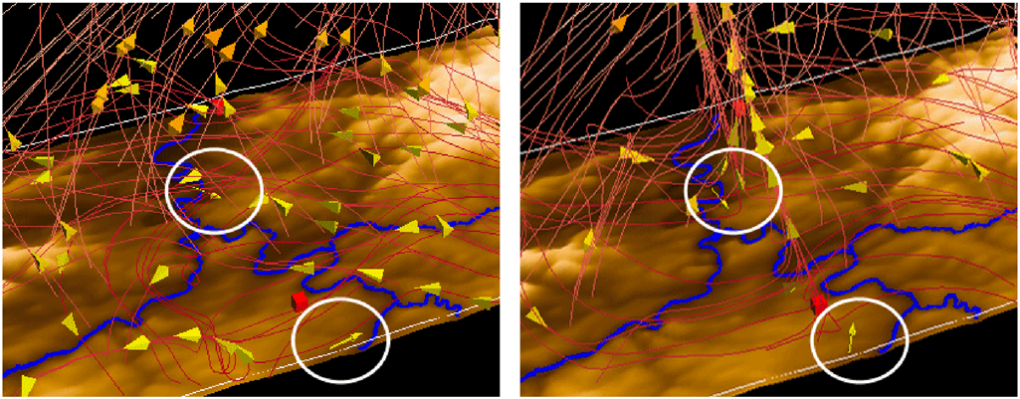
Overlaid view of simulated, observed and static data. Rivers (tubes) and cities (boxes) are displayed for orientation. The simulated wind (streamlines) is visually compared to the observed wind (wind at station represented by an arrow glyph, marked with circles) at 1:00 pm (left) and 2:20 pm (right). Although the comparison of surface station data is a difficult issue, the visualization can be used to get an impression of the level of correlation between the values. (View towards the north)

To get an impression to which extent the simulated wind corresponds to the observed wind, we compare them in an overlaid view. Fig 10 shows the scene at two points in time: 1:00 pm (left) and 2:20 pm (right). In the first one, the wind direction at the stations (marked with circles) point to the opposite direction of the simulated wind in domain 3. On the other hand in the scene at 2:20 pm, the observed and the simulated wind match quite well, pointing to the upstream of the air in the supercell. The observations are only valid at one point in space (the location of the measurement). The simulated value is an averaged value in the model grid box. Furthermore, the static data (orography, land use, soil characteristics) are also interpolated to the grid resolution. Therefore, one cannot expect an exact match of observed and simulated values. This is the more the case, the more the comparison is done in orographic terrain. In general, the comparison of surface station data (especially wind) is a difficult issue since the observations are strongly influenced by the local characteristics of the measurement location (e.g. orography, exposition, land use, soil type). With an increasing resolution of the simulation, the consistencies increase.

**Fig 11:**
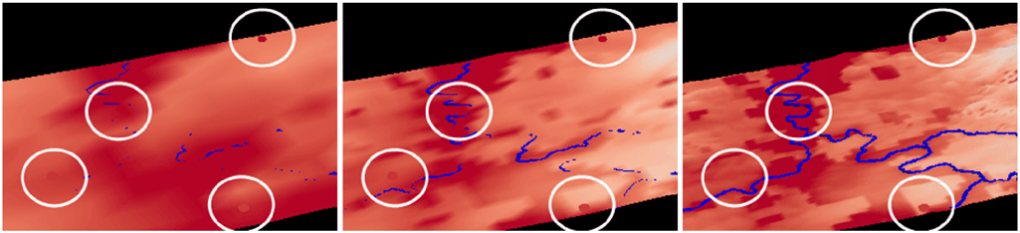
Side-by-side view of simulated, observed data, and static data. The visual comparison of simulated (left to right: 3km, 1km, 333m) and observed temperature (spheres marked with circles) at 2 m at 1:00 pm. The river is occluded in domain 1 because of the finer resolution of the river network (90 m). The refinement of the resolution leads to a better representation of orographic features of the Neckar valley and its confluences. (View towards the north)

In Fig 11, we visually compare the simulated (left to right: 3km, 1km, 333m) and observed temperature at 2 m above ground. It is clearly visible that the river is occluded in the scene with domain 1. The reason is the finer resolution of the river network (90 m) compared to the resolution of the simulation (3 km). Using the same color map for observed and simulated temperature, we can see at first sight that in all domains and at the observation stations, the temperature around the river is higher than in the other regions.

The sequence of images shows the refinement of the resolution and therefore the better representation of orographic features of the Neckar valley and its confluences. Since the valley is deeper in the 333 m simulation, higher surface temperatures are simulated. The temperatures measured in the river valleys are well represented by the model whereas steep slopes in valleys are underestimated even with 333 m resolution. Apart from the warmer river valleys, the model simulation shows warm squared patches. This unrealistic near-surface temperature distribution can be attributed to the coarse grid of the land use data that was used as a boundary condition for the simulation.

#### Task 3: Using the interface by different user groups

To manage task 1 and 2, it is necessary to provide a set of interaction methods to handle time and multirun data as well as to navigate through the case study region (zooming, rotating, and panning). We focus on two different usage scenarios. In the first one, users are scientists, who want to explore and analyze their research results, evaluate their hypotheses, and have discussions with colleagues based on the visualization. In the second scenario, presentation to the public and other scientists is the aim as well as to use it for education. With MEVA, we provide an application, where users are able to easily discover recent research results and playfully explore them. Even inexperienced users find their way to use the application and interpret the data quickly because of the use of intuitive representations of variables (e.g., streamlines for wind, white isosurfaces for clouds) and the limited functionality of the provided GUI.

Our development process is iterative, including the following steps: designing, testing, and prototyping/implementing. Together with experts in visualization, meteorologists, scientists from other domains, and based on our previous work [30], we identified a set of required interaction functionalities, designed the GUI, implemented the functionalities, and finally tested the prototypes with the users. We kept the functionalities to a minimum as described in requirement D.

**Fig 12:**
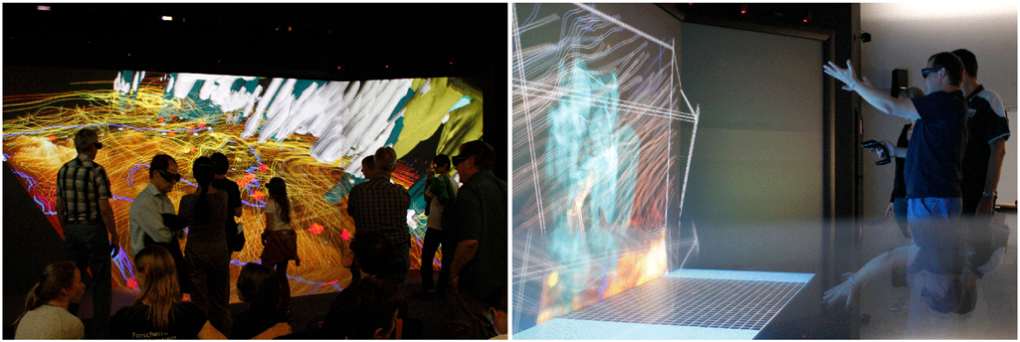
Presentation of the visualization. The interested public discovers the weather simulation results in the TESSIN (Terrestrial Environmental System Simulation and Integration Network) VisLab at the UFZ (left hand side). Thus, the application supports the understanding of complex processes and is a tool for public education. The photo on the right hand side shows a situation in the VisLab where the domain experts use the visualization to analyze their simulation results. (Photos: Lars Bilke)

With our approach, the domain experts were able to analyze the simulation runs and gain new insight as described in task 1 and 2. The interactivity of the visualization helped them to evaluate their results and explore the data to gain new perspectives. To prove our statements concerning our application, we conducted several user studies with different levels of formality. We did interviews with domain experts and presentations for the public in the context of events like open houses (see Fig 12), where we collected feedback and included it in the development process. The test environment was the TESSIN (Terrestrial Environmental System Simulation and Integration Network) VisLab, which was the visualization environment for several visualization projects in the past (see Bilke et al. 2014 [48]). The application was also tested on PC, 3D projector and head-mounted display. After a brief introduction, the users were able to navigate through the visualization and use the given interaction methods to control and manipulate the scene.

To evaluate the final version of MEVA, we conducted a user study. Therefore, we organized a series of workshops in the VisLab. The 16 participants were from various scientific disciplines ranging from meteorology to mathematics to computer science. Because of the different backgrounds of the participants, not all of them had experience in meteorology (44%), with VR environments (50%), and with other 3D devices (38%). The users got a short introduction about the background of the underlying data and the interaction concept of the VisLab. They were told to solve two tasks with the help of the application: (1) compare the observed and simulated temperature; (2) find differences in the simulation runs with varying horizontal resolutions. With the help of a questionnaire, their feedback was collected. In the following, we present the outcomes of the study. As we gave our participants the freedom to skip questions, the number of answers for each question varies between 11 and 16.

We asked the participants if they would use the visualization application for their own data, and they stated that they would find it useful for presentation (94%), exploration (81%), and analysis (88%). In addition, some participants proposed some more fields of application such as teaching and discussions.

For 83% of the participants, it was easy to solve the given tasks after a short introduction, which was described as appropriate by all of the participants. They evaluated the usability as good or very good. The menu is intuitive (79%), gives the user control over the visualization scenes (86%), and the participants were able to correct erroneous input (92%). We also asked if there was a demand to further customize options for the menu (e.g., change font size, assignment of keys), but just 23% saw a need to have these options.

We provided a gamepad as the interaction device. In former tests, the participants used a flystick, which is a common device in VR environments. We observed that it is hard for most participants to become familiar with this device, which most of them never used before. The result of the user test with the gamepad was that 91% had no problems with the navigation. This result confirmed our impressions and led to the decision to support this device in future applications.

The representations of the variables were evaluated as intuitive by 80% of the participants. The majority, 93% of the participants was able to compare different simulations easily and could discover correlations between variables. Also, 69% of the participants state that they learned something new with the help of the visualization (e.g., about the circulation in a supercell and its structure).

With respect to the task of comparing the simulation data with observations, just 63% state that they were able to compare them. The reason was not the application itself, but the lack of observation data that was provided. For a better comparison, more observation data should be included (e.g., rain from radar data).

To sum up, the user study showed us that there is a large interest in MEVA. The participants want to use MEVA for their own work in the future. The provided data is a very important basis for a meaningful visualization. The participants from all scientific domains and with various experiences with 3D devices were able to get along with the application very quickly. A big advantage of using a VR environment as presentation device is that it attracts people and offers good preconditions to stimulate interdisciplinary discussions and productive work sessions (cf. Bohrer et al. 2008 [49]).

#### Task 4: Viewing the presentation on different devices

In scientific projects it is common that cooperation partners are regionally distributed. In our case study, the domain experts are from different areas in Germany. Therefore, it is necessary to provide the visualization for different devices, ranging from common PCs to 3D devices like virtual reality environments, 3D projectors, and head-mounted displays. The use of high-resolution virtual reality environments enables the users to conduct collaborative, interactive walkthroughs, where they can explore their data in detail and navigate through the visualization while discussing and sharing their discoveries and thoughts.

We implemented an input device setup that is usable with a computer mouse, joystick, flystick (commonly used in VR environments), and gamepad. To reach a wide audience, it was necessary to support multiple platforms. The game engine Unity does that out of the box. We successfully tested our visualization with the virtual reality environment TESSIN VisLab (see Fig 12), a head-mounted display, a 3D projector, and several PCs with different hardware.

## Conclusions and future work

We have presented a workflow and application to explore, analyze, and present multifaceted meteorological data, at different spatial and temporal resolutions, from multiple sources (various simulation runs, observations, and static data) and including numerous variables. The workflow leads from input data to an interactive 3D visualization using advanced representations for the variables. To provide interaction for the user, we designed and implemented modular and reusable interfaces for the game engine Unity. We presented two different solutions for multirun data: side-by-side and overlaid comparison. Furthermore, we developed an easy-to-use interface in an iterative development process. We defined two usage scenarios for the application. The first one addresses domain experts and is used to analyze and explore their data as well as to evaluate hypotheses and facilitate discussions. The second one addresses presentation issues and is used to present research results to project partners, decision makers, and the interested public. To prove our statements concerning our application, we conducted several user studies with different levels of formality. Besides interviews with domain experts and presentations for the public, we conducted a user study with 16 participants to evaluate the final version of MEVA.

The novelty of our approach is to provide a flexible workflow that consists of software components and supports researchers of various disciplines to generate meaningful visualizations. Our aim was not to develop new algorithms and techniques, but to provide a toolbox that combines these existing techniques in a useful way (cf. Sedlmair et al 2012 [50]). By utilizing the general purpose visualization system Paraview as initial part of our workflow, MEVA can easily be used also in other application areas.

The analysis and comparison of the simulated circulations and mass fraction in a supercell with different horizontal resolutions (task 1) provided an interesting insight into the process chain in a developing supercell and allowed detailed verification of the model’s representation of this hazardous meteorological phenomenon. This allows the further improvement of the model through the tuning and refinement of the model physics. Moreover, the visualization approach is predestined to improve the understanding of the processes taking place in nature.

With the help of the user study we could find out that the comparison of observation and simulation data (task 2) using MEVA was an easy task for the participants. But we also got the feedback that the participants found the observational data too sparse for a meaningful visual comparison. To solve that, additional observational data from other sources might be included, such as rain radar or satellite images. This could be done using the flexible workflow we developed: after preprocessing, the data could be visualized with ParaView and then added to MEVA, where it would appear as an additional item in the objects menu.

The user study showed us that the participants from various scientific domains were able to learn using the application very quickly (task 3). Even participants with little know-how in meteorology and no experience with VR environments were enabled to explore the data. Most of the participants could learn something new with the help of the visualization and want to use MEVA for their own work in the future.

MEVA was developed to be usable with different devices (task 4). The option of the presentation on a PC was very important during the development process because the project partners are situated in different parts of Germany. Thereby the current versions of MEVA could be tested regularly. The option to run it on a PC is also required for education purposes to make it usable by lecturers and students. For detailed analysis of the data, the use of MEVA in a VR environment was necessary to examine complex structures especially in the simulated wind circulation. During our user study in the VisLab we could observe that the participant’s attention was caught very quickly and discussions have been stimulated while using MEVA.

The growing importance of visualization in many scientific domains caused by an increasing generation of complex, large data sets is apparent. With the development of a flexible workflow, the basis for the generation of visualizations for other application domains is provided. The use of existing software applications, which are enhanced and updated regularly, saves own development effort. Thereby even new and modified data types can be handled easily. The user study showed us that there is a large interest in MEVA and, with our approach to use freely available software as components of the workflow and provide the implemented scripts and example data, it is accessible by many users.

We believe that MEVA will be the basis for other visualization projects in environmental science, for example to compare climate scenarios, simulation results of groundwater and geological models, and for multimodal scenarios, which include multiple models with interacting parts. In the future, we would like to develop an automated parallelized workflow, which would then allow in-situ visualizations in high performance computing environments (cf. Olbrich 2000 [51]).
